# I am done with this! Women dropping out of engineering majors

**DOI:** 10.3389/fpsyg.2022.918439

**Published:** 2022-08-12

**Authors:** Susana González-Pérez, Miryam Martínez-Martínez, Virginia Rey-Paredes, Eva Cifre

**Affiliations:** ^1^Department of Business Economics, School of Economics and Business, Universidad San Pablo-CEU, CEU Universities, Madrid, Spain; ^2^Department of Economics, School of Economics and Business, Universidad San Pablo-CEU, CEU Universities, Madrid, Spain; ^3^Department of Developmental, Educational, Social Psychology and Methodology, Faculty of Health Science, Universitat Jaume I, Castellón, Spain

**Keywords:** STEM, self-efficacy, belongingness, motivation, curriculum, gender stereotypes, persistence

## Abstract

Women are still underrepresented in STEM careers (Science, Technology, Engineering, and Mathematics). One of the possible drivers behind this gender gap in the labour market is the female dropout from STEM education. The causes of the gender differences in the persistence of pursuing STEM studies have been explained by multiple factors related to interest and resolution in this type of career. The goal of the present research is to study the Engineering persistence gender gap in higher education by exploring the main factors underlying the leakage in the pipeline of Engineering fields. Our study reports the results of 34 qualitative in-depth interviews where internal barriers, stereotypes and external obstacles are assessed by women who have left their university degrees, compared with men who have withdrawn and women who have persisted. Results from the content analysis suggest that the undermining of persistence in Engineering fields is related to factors such as the chilly and hostile environment in classes or the workload from an excessively demanding curriculum. Other factors affecting women’s withdrawal are the lack of role models and the perceived incongruity between the female gender role and STEM roles in society, leading to a weakening of female students’ self-efficacy and eroding their sense of belongingness, even making them consider dropping out of their Engineering degree. These findings provide information for the design of future STEM interventions aimed to enhance women’s persistence in STEM university studies.

## Introduction

The need for Science, Technology, Engineering, and Mathematics (STEM) development to increase and maintain our current quality of life is globally acknowledged. STEM fields are the basis of our everyday lives, being responsible, for instance, for having clean water, food to eat and life-saving medicines. However, most of these advances have historically been seen as male domains, with a clear need to extend them to women. According to Eurostat (2021), in 2019, out of a total of 15.4 million posts in Science & Engineering (including physicists, mathematicians, life science professionals, and engineers), there were only 6.3 million female scientists and engineers accounting for 41% of all employment in the European Union. In the OECD countries, the percentage of graduates in Engineering reaches 14%, however, the composition is very different between men and women. Among men, the percentage reaches 25%, while among women only 7%. A situation is very similar to that found in Spain, where women in engineering are 6% compared to 22% of men. However, there are OECD countries where this situation has been mitigated, as is the case of Iceland (OECD, 2019). STEM fields are not only essential to improve our quality of life; they are expected to grow by 10.5% between 2020 and 2030 in the United States (US Bureau of Labor Statistics, 2021). Currently, the top 25 college degrees by pay and demand are all in STEM subjects (World Economic Forum, 2021), mainly in male-dominated jobs (i.e., top 5: Architectural Engineering, Construction Services, Computer Engineering, Aerospace Engineering, and Transportation Sciences and Technologies). Despite this, a large number of STEM undergraduates drop out of their fields. Compared with non-STEM studies, STEM drop-out is in general higher; this is even more marked in the case of women, with female students displaying a 23% higher drop-out rate than their male counterparts, even though these female students in STEM appear to be positively selected in terms of study capital (Isphording and Qendrai, 2019). Barriers to entry into STEM education for female students have gained strong attention from education researchers, such as gender stereotypes (i.e., Master et al., 2016), prior achievement and attitudes (Marsh et al., 2019), or in general, social factors, institutional structures, poor advice and early education environments (Blackburn, 2017). However, subsequent gender differences in continuing to pursue STEM studies are less well studied. Even interventions performed to improve graduation rates for students in STEM (i.e., Hamm et al., 2020) do not consider gender a core issue (just as a control variable). Among all the STEM disciplines, Engineering is one of the most male-dominated due to its well-recognized difficulty (Center of Research and Educational Documentation and Women’s Institute, 1988; López-Sáez et al., 2011). Research in higher education indicates that there are many factors that influence female students’ retention in Engineering (Blickenstaff, 2005; Eddy and Brownell, 2016), contributing to perpetuating the gender gap in this type of major. The lack of women in Engineering is a concern shared by public and educational institutions (Chavatzia, 2017) Despite such increasing efforts to find solutions, we continue to struggle to understand the reasons that lead women to leave Engineering degrees (Beasley and Fischer, 2012; Makarem and Wang, 2020). This study aims to tackle the gender gap in progression in Engineering majors, drawing on two theoretical approaches from both motivation and gender studies through a mixed qualitative-quantitative methodology that will allow delving into the Engineering drop-out phenomenon for female students. Thus, this study aims to address this gap by examining the reasons why female students withdraw from Engineering studies, and to find effective interventions to improve retention rates.

Summing up, this paper contributes to the literature through three main points:

1. Analyzing gender differences in drop out from Engineering majors, but also comparing with female students who continue their studies.
2. Giving a voice to female students who have dropped out of such careers, using the in-depth interview technique to listen to their personal experiences.
3. Providing outcomes that will facilitate the design of adjusted interventions. Understanding the reasons for dropping out will help to devise more effective and efficient strategies for women to stay in STEM careers.

### Engineering dropout: Student motivation and the Tinto’s persistence model

Students do not seek to be retained, they seek to persist (Tinto, 2015). Students’ persistence could not happen without proper motivation, as Engineering students are faced with a very challenging curriculum (Wang and Degol, 2013; Kelly, 2016) and need to expend a huge effort to succeed in their studies. To increase completion rates, institutions are required to adopt a student perspective, to understand how students’ motivation is shaped and which measures or interventions can be addressed to enhance this motivation. When adopting this student perspective, they must do so taking into account that the reality of women and men is not the same, i.e., adopting a gender perspective. According to the European Institute for Gender Equality (EIGE) (n.d.), this means taking gender-based differences into account when looking at any social phenomenon, policy, or process. We will base our study on two different but complementary theories: one that comes from the field of education (Tinto, 1975, 2015) and another from gender studies (social role theory, Eagly, 1987a) to explain and promote the persistence of female students in Engineering majors. Tinto’s model is a conceptually useful framework to analyze student dropout since it reflects the process that an undergraduate student experiences between the decision to abandon or continue their studies. This scheme is based on the fact that in order to continue and be successful, students must integrate socially and academically into the university. Thus, Tinto integrates the academic and social perspectives and ties them together to explain student dropout. The social role theory argues that the proximal causes of sex differences in individual behavior are framed by gender roles or the shared beliefs that apply to individuals based on their socially identified sex (Eagly, 1987a,b; Eagly et al., 2004). Because in all cultures women and men tend to specialize in different behaviors, people have different beliefs about what each sex can and should do, i.e., gender roles are descriptive and prescriptive (Wood and Eagly, 2010). These beliefs constitute socially shared stereotypes within a society, meaning that gender roles are reflected in a society’s stereotypes about men and women. As Wood and Eagly (2010) point out, the descriptive aspect of gender roles indicates what is typical for each sex, so people rely on this information when they are concerned about what is normal for their sex. On the other side, the prescriptive aspect describes what is desirable and admirable for ache sex, so people rely on this information when are motivated to gain social approval or to bolster their own esteem. Thus, in the case of Engineering, women might feel that they are not accomplishing their gender social role of nurturing and they do not identify that Engineering may lead to fulfilling communal goals. In this way, social role theory in general, and the (communal) goal congruity perspective (Diekman and Steinberg, 2013) in particular, will work as a gender mainstreaming theory while developing research questions related to Tinto’s persistence theory to understand how women’s entry, engage and exit of a specific social role (i.e., STEM career).

To be motivated means to be moved to do something (Ryan and Deci, 2000). It is a factor that leads behavior and determines its direction, force, and insistence (Sevinc et al., 2011). Also, motivation is a theoretical concept that is used to explain the beginning, direction, force, and insistence of goal-oriented behavior (Brophy, 2004). This insistence on goal-oriented behavior or persistence is a manifestation of motivation (Bandura, 1989). However, whereas early experiences and goals can lead students to choose Engineering, this motivation changes over time, as students face different college experiences that may affect their willingness to persist. Tinto (2015) proposed that persistence is driven by motivation, which is determined by the lower-order factors of self-efficacy perception, sense of belonging, perceived worth, and relevance of the curriculum. At the heart of Tinto’s model, there is the idea that to be successful, and therefore persist, a student must be well integrated both socially and academically into the college system (Tinto, 1975, 1987). Nevertheless, this integration does not come only from the student but also from the educational environment. In this sense, the social and academic factors linked to the educational environment can help or hinder the integration of students (Casad et al., 2019). This is a useful framework within which not only to investigate the process of student attrition and persistence in Engineering but also to identify possible interventions to reduce withdrawal. Tinto’s perspective requires a holistic approach to studying dropouts taking in different kinds of factors, highlighting that this withdrawal is a process in which it is possible to act (Tinto, 1998). Tinto’s model remains one of the most widely used and cited models in understanding and explaining students’ dropouts (Braxton and Hirschy, 2004; Keup, 2005; Bensimon, 2007; D’Amico et al., 2014), also regarding those in STEM disciplines (i.e., Nicoletti, 2019; Johnson et al., 2020). In particular, Botanga et al. (2021) found in a sample of underrepresented students minority (URM) of STEM that Tinto’s model focuses on integration and belonging, but fails to theorize concepts related to student agency, racial identity, and racism, so important in this URM group. These results stress the need to adapt the model to different samples, such as we do in the case of women in STEM.

### Engineering dropout: The persistence model from a gender perspective

**Figure 1:**
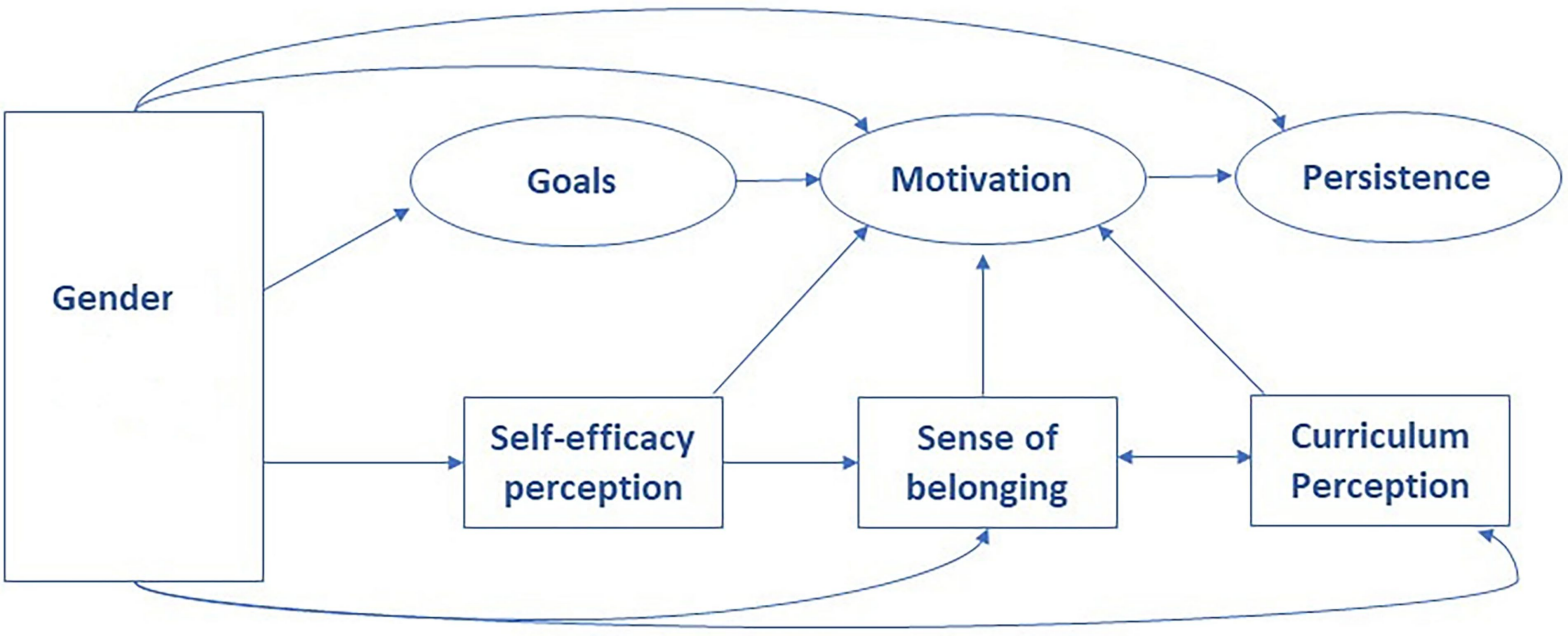
The proposed theoretical model of gendered role congruity in students’ motivation and persistence in engineering.

The social role theory (Eagly, 1987a) offers a framework to understand how features of social roles intersect with individuals’ goals and role pursuits in general, and of women in STEM in particular. Therefore, our research adapts Tinto’s model of student motivation and persistence (Tinto, 2015) considering gender role congruity, so, student’s circumstances and inputs should be included, incorporating their perspective on the main barriers and obstacles that might have undermined their motivation and their willingness to persist in their academic trajectories from a gender perspective (Figure 1).

Tinto (1987) posited that a departure decision was a process by which a student who experienced lack of motivation decided not to persist with college studies. One of the first and more important input variables to determine students’ motivation are goals. According to Locke and Latham’s (1990) of goal setting theory, a goal is defined as what the individual is consciously trying to do. Goals direct attention and action. Furthermore, challenging goals mobilize energy and lead to higher effort, motivating people to develop strategies that will enable them to perform at the required goal levels. Also, the goal accomplishment can lead to satisfaction and further motivation whereas their non-accomplishment can lead to frustration and lower motivation. Goals are shaped by early experiences (Tinto, 1975) as they help specify the orientations the student brings into the college. Goals are a very complex issue and should be approached as a multidimensional variable. Tinto (1998) identified family background, prior schooling and skills and abilities as the main factors shaping students’ goals. In this line, the goal congruity perspective (Diekman and Steinberg, 2013; Diekman et al., 2017) may help to explain why women enter, engage in and exit STEM pursuits, as it provides a framework to understand how motives influence social role selection, and in turn how these social roles afford or impede the pursuit of goals. This perspective is based on the social role theory, which posits that sex differences in individual behaviour are framed by gender roles, or the shared beliefs that apply to individuals on the basis of their socially identified sex (Eagly, 1987a; Eagly et al., 2004). For both sexes, good fit to the opportunities afforded by their society yields rewards in terms of ease of completing important tasks and building satisfying interpersonal relationships, so individuals thus (consciously or not) are more likely to seek and attain the goals that are afforded by their roles (Diekman and Eagly, 2008). According to the goal congruity framework, an important aspect of STEM decisions is the belief that STEM careers do not fulfil communal, other-oriented goals (Diekman and Steinberg, 2013), which is not aligned (goal incongruity) with women’s roles. So, these internalized values tend to drive female students away from male-stereotypic careers perceived lower in communion (Diekman et al., 2010), leading to horizontal segregation (Eagly, 1987b; Wood and Eagly, 2012). Also, as proposed by Tinto’s model, the (communal) goal congruity considers the temporal dimension in three phases: (1) anticipate (in)congruity prior to role decisions, (2) experienced (in)congruity in a particular role, and (3) psychological and behavioral responses to maintain or seek congruity (Diekman et al., 2017). So, the socialization process plays a key role, as societal gender stereotypes (Bakan, 1966) lead even young females (Block et al., 2018) to internalize communal values instead of agentic traits (Eagly, 1987b) which are not congruent with the expected agentic traits associated to STEM. This anticipated goal incongruity may fuel the decision not to prior enroll in a STEAM career. The second phase focuses on what happens after individuals enter into social roles, i.e., STEM majors. Beliefs about anticipated goal (in)congruity might be more or less accurate with their actual experiences of goal (in)congruity. Then, in phase 3, individuals respond to maintain/seek congruity. To do so, they might change the motives (i.e., downplaying the importance of the motive, such as communality) or roles (i.e., dropping out STEM majors).

#### Motivation: Self-efficacy perception perspective

Among the other lower-order factors in Tinto’s model that determine motivation, self-efficacy is especially important. Self-efficacy is an aspect of social cognitive theory defined as “the exercise of human agency through people’s beliefs in their capabilities to produce desired effects by their actions” (Bandura, 1997) or “judgments of how well one can execute courses of action required to deal with prospective situations” (Bandura, 1982).

In this line, prior schooling and preferences for school subjects strongly influence whether women feel motivated to study STEM at college (Delaney and Devereux, 2019) based on their experience. Self-efficacy has been shown to mediate perseverance, as students who have higher self-efficacy are more likely to persist in the face of difficulty (Seymour and Hewitt, 1997; Zimmerman, 2000; Usher and Pajares, 2008). Regarding the sources of self-efficacy, this could be built by mastery experience in the classroom, i.e., by succeeding in a perceived to be very difficult exam or assignment (Usher and Pajares, 2008). Thus, the role of school subjects in later STEM enrolment contributes to the gender gap in STEM in college, making it critical to provide positive STEM experiences in school to increase female students’ interest in STEM fields (Fervers et al., 2020). Skills and abilities also shape female students’ goals, motivating them to enroll in a STEM major, for example, spatial skills (Halpern et al., 2007) and perceptions of ability have been found to predict career choices in Engineering (Eccles and Wigfield, 2002). Emotional or physiological states are also sources of self-efficacy that students may feel when completing a difficult task successfully (Phan, 2012; Phan and Ngu, 2016). Social persuasion (Bandura, 1997) is the external encouragement received from peers or faculty members. Finally, vicarious experience frequently occurs for students when one compares oneself with another peer (Bandura, 1997). In this line, sociologists have identified the so-called occupational inheritance phenomenon (Mannon and Schreuders, 2007), which shows that female students entering Engineering are more likely than men to have an engineer in the family, while those without engineers in the family must find another figure to inspire and motivate them. In sum, family, especially parents, are critical early socializers of their children’s academic interests and their academic choices of STEM majors (Simpkins et al., 2006). Nevertheless, having the possibility to interact with role models (such as an engineer in the family or an inspiring science teacher) reduces the effect of gender stereotypes and increases intentions of female students’ enrolment in STEM majors (González-Pérez et al., 2020). Also, intervention programs focused on self-efficacy sources have shown to be greatly successful to increase interest towards STEAM (STEAM + Arts) in female students (Ofori-Boadu, 2018).

#### Motivation: Sense of belongingness perspective

The third construct of Tinto’s model, belongingness, has been linked with persistence at university (Webb et al., 2017). It is understood to be the sense of connectedness an individual experience within the learning environment (Osterman, 2000). In other words, belonging refers to students’ sense of being accepted, valued, included, and encouraged by both teachers and peers and of feeling an important part of the group (Goodenow, 1993). The literature suggests that there is a strong relationship between the concept of belongingness and students’ self-efficacy perception (St-Amand et al., 2017). The quality of belongingness is dependent on a variety of factors such as the level of a student’s involvement in the different activities provided by college and the availability of support, which finally builds a sense of connection (Picciano, 2002). If these female students feel connected it will be more likely that they will be receptive and more deeply engage in learning (Roxburgh, 2012), while if female students experience greater uncertainty and feelings of not belonging, unsure of their social bonds and sensitive to rejection, they are at more risk of dropping out (Walton et al., 2015). There has been much research around the structural barriers that women face in STEM fields and that make them feel like ‘strangers’ in science (Massachusetts Institute of Technology, 1999; Sonnert et al., 2007).

Classroom climate affects students’ belongingness perception and therefore their motivation and persistence in STEM (Shapiro and Sax, 2011). If female students in STEM face a difficult climate in the classroom, it could disproportionately affect them, producing feelings of depression and lowering self-confidence (Strenta et al., 1994). Women entering a male-dominated field may need to face social marginalization and may experience a climate in which they may feel unwelcome (Flam, 1991). Colbeck et al. (2001) suggest that this “chilly climate” for women in Engineering results from peer interactions, which is especially relevant as peer acceptance is a central concern in adolescence (Eaton et al., 1991). While it is perceived that peer interactions affect students more than faculty interactions, teachers also have an important role in making students feel comfortable and accepted in college, as well as promoting students’ interest in their subjects (Astin and Sax, 1996).

Furthermore, women are more likely to leave STEM majors compared to men, in part because they lack similar role models such as teaching assistants and instructors (Marx and Roman, 2002). Women exhibit a self-perception of belonging in STEM culture and are more motivated to pursue studies in the presence of female role models (Stout et al., 2011). Nevertheless, men comprise the majority of STEM faculties that may not only signal that women do not belong or cannot succeed in these fields (Walton and Cohen, 2007) but also gives female students limited access to female faculty role models. In this context, with a limited representation of female students, even highly skilled and motivated women may wonder whether they belong on STEM major programs (Cheryan et al., 2009), which elicits that a more inclusive Engineering community is a crucial element so that female students do not feel alone (Ayre et al., 2013). However, faculty can also be a threat to female persistence in STEM fields, because as Hall and Sandler (1982) explain, faculty interactions can dampen women’s ambitions, especially in male-dominated fields such as Engineering. Furthermore, researchers such as Wasburn and Miller (2004, 2005) have found that faculties treated male and female students differently, as they tend to be more condescending, and less respectful to female students. They have also found that faculties tend to exclude women from certain activities, for example giving them menial tasks within group projects. Grading criteria are often found unfair and biased, especially as female students feel they need to excel more than their male counterparts (Seymour and Hewitt, 1997). These authors found as well that ignoring, or tolerating misogynism, feel female students unwelcome in class. In both cases, both male-peer and faculty interaction might be biased due again to the goal and trait incongruence that they perceive between the female (communal) traits and what is thought to be successful for a masculinized career (agentic), which might in turn lead to prejudice against women (see for instance Eagly and Karau, 2002, about women leaders, i.e., in a masculinized career). Consequently, women might divert from STEM pathways because of gender stereotypes and prejudice (Diekman et al., 2017). For instance, both male and female science faculty have shown gender bias in preferring male over female applicants for a lab manager position, even when qualifications were matched experimentally (Moss-Racusin et al., 2012).

#### Motivation: Curriculum perception perspective

Curriculum, the last element of Tinto’s model explaining motivation, is explained in turn and affected by the sense of belonging (Webb et al., 2017). Curriculum needs to be understood in an extended way, considering not only what is being taught (Kelly, 2009), but also the methods of assessment. Notably, students in STEM fields often earn lower grades than students in other fields (Ma and Liu, 2015). This is another factor that hindered female students studying for a degree in Engineering, as perception of low grades seems to be more discouraging for them than for their male counterparts (Lent et al., 2002). Moreover, the usage of curve-grading assignments encourages competition, where male students tend to feel significantly more comfortable, preventing cooperation and peer support (Guzdial et al., 2001).

Griffith (2010) found that students feel that classes in Engineering tend to be boring and needlessly difficult, forcing them to spend many hours and make huge efforts, sometimes without the desired outcome. This situation affects female students to a greater extent, undermining their perception of belonging and leading to them opting to drop out to a greater extent than their male counterparts (Seymour, 1995). Seron et al. (2018) discovered that during the first 2 years of Engineering majors, it would be necessary to incorporate as many real, everyday examples, as possible to continue to encourage women students to stick with challenging introductory classes (Wang and Degol, 2013; Kelly, 2016). In terms of the type of teaching methods, hands-on projects are more meaningful and interesting for female students (Mitchell, 1993; Halpern, 2004; Geist and King, 2008). Project-based learning (Blumenfeld et al., 2000) has proven to increase students’ engagement as well as a deeper understanding of scientific problems (Kaldaras et al., 2021). Instruction based on memorizing without understanding has become obsolete. A better and deeper understanding of science enables students to explain phenomena and solve real-life problems, while engaging more female students (Wan, 2021). Collaborative projects and environments are particularly helpful for female students (Wang, 2012), as it can provide them with real-world applications of science, reinforcing their decision to persist (Margolis et al., 2000). When faculties embrace these real-life situation teaching techniques, female students’ learning and confidence levels improve (Hyde and Gess-Newsome, 2000), nevertheless STEM majors have not yet fully embraced these more collaborative teaching styles (Laird et al., 2007). However, it is noteworthy to remark that, contrary to expected, recent studies (i.e., Sax et al., 2018) have found that feeling supported by the computing department, as well as by peers, results to be central to fostering women’s and minority students’ sense of belonging in the field of computing, even more than specific inclusive pedagogical practices.

### The present study

While most of the existing studies addressing these topics have been conducted using mostly quantitative methods (Arriaga et al., 2011; Bernardo et al., 2017), there is a lack of qualitative research providing in-depth analysis of the views of female drop-out students. When so, although they present interesting research contributions, they have some shortcomings. This is the case of the one of Casanova et al. (2021), which is not focused on the attrition gender perspective. Others such as Chou and Chen (2015) give an extensive vision of current engineering female students’ perceptions, but without pointing out the divergences between the ones who persist and the ones who switch or drop out. Even Madara and Cherotich (2016), who studied the challenges that face female engineering students, highlighted just the perspective provided by current students. The present study aims to give voice to female dropout students to seek the underlying reasons that lead them to leave their majors. Dropout and non-dropout female engineering students face similar challenges in male-dominated majors, however, there are triggers that make them decide to switch, while others decide to stay. Understanding how women who continue in engineering do differ from those who leave could help us to find useful individual and organizational tools for helping them to stay. On the other hand, we aim to get a better understanding of how female students who drop out feel that their sense of belonging and motivation to persist was undermined in a male-dominated major. Therefore, we propose the following research questions to be answered by in-depth interviews addressing the following research questions:

In what ways do women who drop out have different goals that lead them to consider engineering majors to be less aligned with female (communal) gender roles than women who do not drop out or men who drop out?
What are the main differences found in terms of self-efficacy perception between female and male students who drop out?
In what ways does the sense of belongingness (chilly climate) of students who drop out differ from that of students who do not drop out?
In what way may the perception of the curriculum (collaborative methods and grading system) affect female motivation for persisting in Engineering?
In what ways does role congruity perception impact female students’ attrition rates?
What types of interventions or measures could be taken in order to better prevent female students from abandoning their studies?

## Methods

### Procedure

We used a mixed-methods approach combining qualitative and quantitative analyses. The overall purpose of mixed methods studies is that the use of quantitative and qualitative approaches in combination provides a better understanding of the research problem than either approach alone (Caracelli and Greene, 1993; Creswell and Plano Clark, 2017). We applied qualitative analysis to explore the ideas, behaviors, and feelings of student participants and quantitative methods to determine the direction or extent of these insights. Greene (2011) points out several advantages of mixed methods research: complementarity (the results from one method clarify the findings from the other method), development (the results from one method help to develop the use of the other method, for example, to inform future research) and expansion (in our case, seeking to extend theories about the causes that prevent women from dropping out from STEM degrees).

According to Molina-Azorín (2016), data collection refers to the sequence the researcher uses to collect both quantitative and qualitative data. In this research, we gathered the information at the same time (concurrent design) which means that researchers seek congruent findings. Thus, in the in-depth interviews, we asked participants to quantify some nodes.

### Sample

We performed a non-probability sampling method including quota sampling and snowball sampling. In a quota sampling, researchers develop control categories, or quotas, of population elements whereas, in a snowball sampling, participants are asked to assist researchers in identifying other potential subjects (Malhotra, 2008).

In this study, we conducted 34 in-depth interviews with students participating (or that have participated) in an Engineering major and we posed 3 quotas: 10 dropout male students; 10 dropout female students, and 10 non dropout female students, however, we decided to extend the number of female non-dropout participants to strengthen the recommendations to persist (RQ6). Snowball sampling design was applied to identify potential subjects in each quota. We initially contacted 5 women who were studying for an Engineering degree and asked them to look for other women persisting in Engineering or women or men who have abandoned their Engineering studies. Looking for students who were willing to participate in the research has not been an easy matter, especially for women who drop out of engineering majors because, in most cases, they have assumed it is a personal failure that they find difficult to talk about. Therefore, we reached an agreement with the Royal Academy of Engineering and ASTI Talent and Technology Foundation, which helped us by providing the contact details of women studying engineering. Finally, the sample consisted of 9 male dropout students, 10 female dropout students, and 15 female non-dropout students. Participants were born between 1994 and 2003. All of them started an Engineering major and most of the students who dropped out changed to majors in the social sciences such as business administration, economics, business intelligence, or international relationships. Participants’ characteristics are reported in Supplementary Table 1.

### Study design

The research team designed the study by generating hypotheses about possible causes and associated features that prevent women from dropping out of STEM majors, based on the theoretical model developed in the previous section. This led to the design of a semi-structured interview (Marshall and Rossman, 2014) to understand the factors that influence female students’ retention in engineering. Questions related to the persistence of students in engineering degrees were developed based on previous research on female students in STEM (González-Pérez et al., 2020).

To ensure the objectivity of the interview process, the authors carefully wrote and rewrote all the questions (consulting with outside third-party colleagues) both to improve construct validity and to ensure that the authors did not lead respondents in their answers (Gibbert and Ruigrok, 2010). A common set of questions was presented to all participants in a semi-structured interview to identify both positive and negative experiences that have occurred over the course of the respondents’ academic life. Interviewers established a climate of trust to ensure that respondents felt safe in sharing their experiences. Thus, their experiences, rather than the authors’ perspectives, drove the research.

Participants who agreed to participate in our study were scheduled for an interview with a researcher. Given the sensitive nature of the topics covered in our interviews, interviews began with an explanation of the purpose of the research, a reiteration of the assurance of confidentiality, and an opportunity to allow respondents to ask any questions before starting. All interviews were conducted online by Teams or similar apps. Online interviews, in addition to saving time in commuting, have been shown to produce as reliable information as face-to-face interviews and, in some cases, may even ease respondents’ anxiety (Salmons, 2015).

Each interview lasted half an hour on average, was recorded by the interviewer, transcribed by the research team, and completely anonymized. Data were collected from December 2021 to February 2022.

### Measure

Transcriptions of all the interviews were entered into Nvivo 12 to organize and manage the data. Interview questions focused on the following areas: motivations for choosing an Engineering degree, course design and subjects they feel more comfortable with, self-efficacy perception, belongingness, chilly climate with classmates and/or teachers, and socio-economic status. Indirectly, we looked for the factors underlying the women’s decision to leave engineering degrees/majors, that contribute to the persistence of gender inequalities in STEM fields. The interview guide, based on the research questions and a review of the literature, included the following general questions: (1) What motivations led you to choose an engineering degree? (2) To what extent have you used collaborative projects or with a practical approach? (3) Did you feel at any time that you were not capable of getting an engineering degree? (4) How do you think relationships with peers and teachers influence the decision to persist? (5) Do you consider that socioeconomic status influences to study engineering? Nodes include gender congruity, goals (attitude and early experiences, role models, socio-economics status), self-efficacy, sense of belongingness (chilly climate with classmates and chilly climate with teachers), curriculum perception, and persistence. Nodes are described in Supplementary Table 2.

To homogenize coding methods, four interviews were randomly selected and independently analyzed by three authors to identify the content representative in each node, as well as novel themes. After coding, these three authors discussed the nodes and paragraphs representing them and agreed on node labels and definitions, developing a codebook that facilitated reliability among raters. The remaining 30 transcripts were then coded separately by two authors using the codebook and labelling segments of text according to whether the content appeared to pertain to one or more of the defined nodes.

The two coding authors then compared their individual assessments. The reliability of the coding between the authors resulted in 97.04% agreement. To test this interrater reliability, we obtained the Cohen’s Kappa coefficient, resulting in 0.55. The Kappa coefficient is a quantitative measure of reliability for coders rating the same phenomena, corrected for how often the raters may agree by chance (Cohen, 1960). As Cohen suggests, a Kappa coefficient superior to 0.41 should be acceptable.

To meet the assurances on confidentiality given to participants, the authors did not involve a third party in coding interviews.

According to the quantitative research, we used the Mann–Whitney U test for independent samples (a non-parametric alternative to paired *t*-test) to rate differences in the mean values between male and female students who have dropped out; and between female students who persist and female students who have dropped out.

For this purpose, in the interview, we asked participants questions such as: (1) Do you think that men and women have different motivations to choose a university degree? (2) Did you feel more interested in subjects with a more practical content or a more collaborative approach? (3) Have you felt that you had a low self-efficacy perception? (4) Did you experience a chilly climate with teachers and/or classmates? (5) To what extent do you consider that socioeconomic status is a barrier to studying engineering?

Participants answered to the questions using an 11-point Likert scale, where 0 means that they totally disagree with the question posed and 10 that they totally agree with it. For example, in the question “have you felt that you had a low self-efficacy perception?” a rating of 10 means that he/she agrees with this negative self-view, whereas a rating of 0 refers to the opposite, showing a positive self-concept to finish his/her STEM degrees. Previous scholars have used an 11-point Likert scale to measure these issues (Nicolaidou and Philippou, 2003; Mohd Dzin and Lay, 2021; Hitches et al., 2022) whereas other researchers state that the reliability of scales increases with the number of points used (Scherpenzeel and Saris, 1995; Scherpenzeel, 2002).

Statistical analysis of the data was performed with IBM SPSS (version 27) statistical software for Windows; with a margin in the level of accuracy of 95% and an error level of 5% (statistical significance level of *α* = 0.05).

## Results

This research begins with an exploratory qualitative approach, followed by a quantitative analysis of the preliminary results obtained in the interviews. The use of a mixed-method research plays an important role because results obtained from both qualitative and quantitative methods enrich our understanding of the problems and questions of our research topic (Creswell, 2009; Molina-Azorín, 2016).

### Qualitative analyses

Our study reveals that female students who drop out do not find practice-oriented subjects or collaborative projects in the first course, feel a lower self-efficacy perception than male students, and agree with the idea that Engineering majors fit better with male gender roles much more than non-drop out female students and, sometimes, experience a chilly climate in the classroom.

After each quote, in brackets, we have noted the number of the interview and if it is a male or a female who drops out (OUT) or persists (IN). For example (I19_Female IN), corresponds to interview number 19 which is a female who persists in an Engineering major. The encoding density, for females who drop out, females who persist, and males who drop out, can be appreciated in Supplementary Figures 1–3.

#### Role congruency perception

Female students who remain in these majors think that horizontal gender segregation is something that we, as a society, have overcome. Furthermore, women who did not drop out have found that they can make an impact in society through Engineering.

“It does not have to be like this. Each person has their own goals and motivations and can do anything that he or she wants, right now, in the middle of 2022, in the middle of the 21^st^ century. I believe that everyone can choose what they want to do and visualize themselves in one way or another and choose their path from there, without considering if they are men or women.” (I19_Female IN)

“I believe that, in any profession, there are aspects that can be achieved to make an impact in society… as an engineer, I believe that I can make a real impact in our society and contribute with very good achievements without being a doctor or nurse.” (I08_Female IN)

However, female students who withdraw tend to think that these fields are less aligned with female gender roles, even having chosen them in the first place.

“I think that as women, we are more focused on taking care and worrying about others […] There is a social norm that assumes women must take care of people: disabled, children, or even little brothers or sisters. Family care always falls on us and men can dedicate themselves to reaching success. So yes, I think it has something to do with having different aspirations.” (I29_Female OUT)

“It is true that the vast majority of men are more focused on being successful, and I think that my family and friends assumed that since I did volunteer work, I was going to choose a major related to care, such as social work, and by the time I wanted to change, I found that everyone expected me to quit Engineering.” (I32_Female OUT)

Male students are equally aware of the different roles, aspirations, and goals assigned to men and women. They find that society assumes that the most demanding and competitive careers, such as Engineering, are not attractive to women and will remain in male fields.

“That depends on how much each person has been influenced by the gender roles that have been established. Society will tell you if you are a woman, to become a nurse, for example. And there is ninety-odd percent of nurses, but my brother is a nurse. In other words, it is not to a certain extent what a person really wants, because people are also conditioned by society norms.” (I23_Male OUT)

“Absolutely. There are many studies that corroborate it, women have personality traits like compassion, empathy, and more focus on people. However, men generally tend to focus more on objects and tend to be more competitive and technical. And it will always remain this way. Although they may try to set quotas, men will tend to choose more technical majors, especially Engineering, as women will tend to choose majors whether related to literature, nursing or focused on caring. (…) Women and men have different motivations, of course, from a psychological, biological, and sociological point of view.” (I34_Male OUT)

Having high educated parents (Social Economic Status) positively influenced choices not congruent with gender roles for some students, as mentioned by the following interviewee:

“And with the help of my parents, who are university professors, they helped me clear my head, putting together my concerns, and the things I liked, suggesting that Engineering could be the best choice for me.” (I06_Female IN)

“Both my parents have Law majors, and they told me that it was a very good career choice, but as soon as I told them that I was interested in Physical Engineering they were also delighted and supported me.” (I01_Female IN)

#### Goals

Four female students who persist and four men who have dropped out refer to early experiences in science as a relevant factor for choosing Engineering. None of the female students that withdrew referred to this type of previous experience. On the other hand, six female students who persist in Engineering refer to having been influenced by role models, compared with just one woman and one man who dropped out. Therefore, learning vicariously from role models appears to be a powerful tool resulting in higher motivation for female students to pursue and persist in Engineering.

We find out that women who persist in STEM majors tend to have had early experiences and role models that have helped them to build strong goals and motivation to pursue these studies. These influences seem to have reduced their gender stereotypes and made them feel more aligned with these male-dominated majors and roles.

“Well, since I was a little girl, I have always liked science, I asked for gifts of chemistry sets for Christmas. I have always been good at maths and physics at school … I have been given books on why things happen, and why natural phenomena happen, which also helps a lot to be interested in science. And my family. In my family, there are several engineers, my uncle, my grandfather, my cousins, who have been real role models to me…” (I05_Female IN)

“People always tell me that I should have studied medicine because I am a very curious person and I am always asking what everything means, but I think that my curiosity fits much more with getting an engineering degree because in the end you learn from so many things and explain many realities that we have around us.” (I09_Female IN)

Therefore, having contact with science or role models in the early years appears to help women to reduce their gender stereotypes, allowing them to broaden their horizons and consider other types of majors not necessarily aligned with gender roles.

On the other hand, we find that female students who dropped out of engineering majors usually have not had these early science experiences or the influence of a significant role model. We have found that these women tend to choose these majors based more on agentic values, such as having a better job in the future or earning more money. These goals and motivation could not be sufficient to persist when a difficulty appears, as they are not congruent with what is expected from a woman.

“I thought that Engineering was a career that had many professional opportunities because you can do Engineering and work as an engineer or join a consulting firm and dedicate yourself to the business world because in the end it gives you some maths tools and makes it much easier for you to get into any work (…) And that was what I liked about Engineering, I loved the idea of having an advantage over other candidates who could apply for a job […] I have never seen myself working in the Engineering world, I have always had the business world in my head.” (I28_Female OUT)

“In my case, I correspond to the profile of a person who wants to achieve success in life, not for getting recognition, but on a personal level. In other words, I am a very demanding person, and I knew that I wanted to pursue a career that would be interesting and lead me to have a job that I liked and where I could advance and grow.” (I26_Female OUT)

On the other hand, also men students who have withdrawn from Engineering seem to be guided by agentic values, congruent with their gender roles.

“I was guided by the idea that by studying Engineering you will have more open doors, or you will have a greater variety of possibilities, or even being able to choose more types of paths if you want to change at a given moment.” (I13_Male OUT)

“It was a challenge for me, while other careers did not challenge me at that moment […] I decided to do Engineering because it was starting with the most difficult major, even not having a very clear idea what I wanted, and leaving sometime later to start other paths.” (I25_Male OUT)

It seems that women who stay in Engineering have found intrinsic motivation. However, both women and men who have dropped out mimic male traits based on extrinsic motivation, which does not seem enough for helping them to persist in the major.

#### Self-efficacy perception

Low self-efficacy is one of the strongest barriers that women face. This low self-efficacy keeps women on the back foot in engineering majors, limits their aspirations, and leads them to feel they do not suit them. This feeling of low self-efficacy stands out especially in female students, regardless of whether they have dropped out or not. Failing exams repeatedly, having many tasks and exams to do, and comparing themselves with others are some of the arguments put forward by the participants:

“(…) except for the first exam, what I do is fail and even though you have to dedicate many hours to it, and you have to study a lot, I felt that I did not get ahead (…) Yes, I felt frustrated and compared to my classmates, maybe, I don’t know, I didn’t understand very well why they were getting good marks and I couldn’t.” (I17_Female OUT)

“There comes a time when you have so much pressure, so many things that something in your head tells you ‘I can’t.” (I03_Female IN)

“Sometimes you were very well prepared, and you couldn’t get it because it was a very high level, so, for me sometimes there was a feeling of impotence.” (I08_Female IN)

Sometimes, even though they get good marks, they play this down:

“I have a friend (a female student), for example, who got a very good grade in a subject that she was retaking, and it was like oh, well, since I retook it, well obviously I’m going to be among the best, however it was more than that: she was very good. However, she was always saying that it was because she had retaken it and, well, it was normal.” (I32_Female OUT)

As one of the participants shows, low(er) self-efficacy perception has a strong relation with impostor syndrome, the psychological pattern in which one doubts one’s accomplishments, which makes these women have a persistent internalized fear of being exposed as a ‘fraud’ (Langford and Clance, 1993). This is a self-limiting feeling (de Vries, 1990), very much in line with the role incongruity these women feel (Hernandez Bark et al., 2016) being in a world that belongs to men:

“Impostor syndrome in a woman’s life is inevitable. And more so in a world of men. It’s just that it’s impossible not to feel inferior when you’re also getting into a mess all your life that isn’t your place as such.” (I29_Female OUT)

Maybe all of this is due to the fact that women self-impose higher quality standards. Not only do they have to contend with the pressure of their studies but with their own feelings. Referring to this situation, one participant mentioned the following:

“(…) moreover, we are also generally very, very demanding with ourselves. We always try to give our best.” (I09_Female IN)

Another female student missed more motivation from the university:

“I missed having an encouraging push, that they gave me a vote of confidence. (…) someone who told me: come on XXX, you’re going to do very well (…) I would have liked a greater motivation towards myself to achieve it, I think that although it would have been difficult because it is difficult and I do not deny that, however, if they had given me a greater vote of confidence, maybe I would have got it.” (I26_Female OUT)

However, this feeling is not shared by their male counterparts, even though they have dropped out:

“No, at no time. The truth is that even now (after dropping out) I see myself perfectly qualified to study and graduate in Telecommunications Engineering.” (I22_Male OUT)

“No, I always have the impression that if I managed to focus and get serious about it, I would have got it perfectly.” (I23_Male OUT)

#### Curriculum perception

Harsh competitive grading systems, densely packed curricula, and a lack of teaching for conceptual understanding (Seymour, 1995; Zohar and Sela, 2003) negatively affect women undergraduates in STEM majors. Whereas, hands-on tasks, employing active learning techniques, communal, collaborative learning environments, and teaching an understanding of the social relevance of physics in their everyday worlds have a positive impact on self-efficacy (Jansen et al., 2015).

Interviewees highlight the difficulty of the subjects, the assessment of learning, the overload of work, and the lack of time to do it. Three participants express it in the following way:

“Some subjects are almost impossible, either because they are difficult, or because there is a lot of content within the syllabus, many things to study (…) The assessment is also a hindrance, they set some minimums to pass, and it is very complicated... (…) there is a lot of theory and a lot of volume for a short period of time.” (I03_Female IN)

“The Bologna plan places great emphasis on doing many things throughout the year (…). You also must slow down at some point. I always had exams or homework, and I think that sometimes that was quite problematic, because you are always overwhelmed, you always have things to do.” (I32_Female OUT)

Indeed, this situation led the participants to a lack of motivation because they were unable to adapt. There are numerous examples of drop out men and women who described their experience in the following ways:

“The pressure with this new way of studying, was difficult for me.” (I20_Female OUT)

“One of the reasons why I got frustrated with the degree was because (studying telecommunications engineering), during the first year, we did all the programming exams on paper (he refers to not using computers).” (I32_Male OUT)

Another male student goes on saying:

“You lose motivation because it is not oriented to the real world. (…) what I found most was how abstract and outdated I saw the ways of teaching.” (I25_Male OUT)

Since in the first courses there are hardly any collaborative projects or practice-oriented subjects, women who persist especially value this type of learning:

“Practical activities help you to get an idea of what work is like after leaving university. And obviously that helps a lot to motivate you to do your best.” (I07_Female IN)

“Collaborative work has been useful to deepen and put into practice what we have studied (…) it gives more meaning to work…I’m doing it for a reason… I am fighting for something.” (I09_Female IN)

While another female participant who dropped out, posits:

“In the last courses I felt more comfortable (…) since we had a lot of practice-oriented subjects, a lot of laboratories… thanks to those collaborative environments in small groups I had a closer relationship with lectures.” (I32_Female OUT)

Considering the methods of assessment, as a key part of the curriculum, we found that there were mentions about low grades by seven female students who stayed in Engineering, compared with six who dropped out. Their male counterparts who dropped out from the major seemed to be less affected by this low grading as none of them referred to it as a barrier. It could be explained by women’s double standards, as they judge themselves more rigorously about grades. Women entering Engineering tend to be overachievers, who have had the best grades in high school. This can cause a sense of failure and of being out of place, affecting belongingness and even self-efficacy perception. Nevertheless, it does not seem to be something specific to female students who drop out, it appears to be a barrier that needs to be overcome by female students in general.

“Sometimes you were very well prepared, however you could not pass because they asked for an unattainable level, so, I sometimes had a feeling of impotence.” (I08_Female IN)

“I had a very bad time, June of last year was one of the worst moments I’ve ever experienced. Because I have never tried so hard to be able to get something without any success. […] Your exam is shit? I have been told that many times, many times. And my tears were falling, because you cannot tell me that my exam is shit because you do not know the work behind the exam. You do not know the work behind your zero …” (I26_Female OUT)

However, an assessment method that encourages competition over collaboration is a system where female students do not feel comfortable, as stated by interviewees:

“And you were surrounded by guys that … were really competitive, and I felt … a bad vibe, because that doesn’t work for me at all.” (I09_Female IN)

“Men tend to be more aggressive, and competitive (…) And I think that it is because they are more used to competing, not just academically, but in life.” (I20_Female OUT)

Finally, when students were asked about their study and personal life balance, this barrier was mentioned mostly by drop out students (male and female), it looks like female students who have persisted have been able to find some kind of balance:

“Having a life while you’re in college seems very complicated to me… the amount of time you need to study was disproportionate, I felt … overwhelmed, without time for my own life, family and friends. It seems that it is like a part of your life was missing. In other words, it’s like focusing all the time on studying, because if you don’t, you won’t get there… maybe it had to be more years… instead of you spending your entire life studying.” (I14_Female OUT)

“I am very well organized. I have to relax and have free time, maybe I’ll start studying soon and then I make plans, because if I am studying all day it won’t work for me. […] And yes, I think that you need to organize yourself well and to study your daily hours, your 5 daily hours.” (I31_Female IN)

#### Sense of belongingness

Women can experience a chilly climate with teachers or with their classmates. Chilly climate stands out as a barrier which can block the route to their degrees, including feelings of isolation and intimidation, sexual harassment as well as a loss in self-confidence as they progressed through their major program (Blickenstaff, 2005). Female students generally receive less attention from teachers than their male counterparts regardless of the subject or age of students (Wilkinson and Marrett, 1985; Sadker and Sadker, 1994). Furthermore, student–teacher interactions are qualitatively different for male and female students as well, while women ask more questions than men, teachers give them less feedback (Spear, 1984; Eccles and Blumenfeld, 1985; Sadker and Sadker, 1994). Four participants express this situation in the following way:

“There was a professor who …, it was my second enrolment in Calculus, I went to review my exam …. and professors were quite old in general … so, he saw me, a woman having failed calculus for the second time and told me that I wasn’t suitable for Engineering. And in the end it takes you down. And I even considered leaving Engineering and studying something else that has nothing to do with Engineering, because they have told me precisely that I was not suitable.” (I02_Female IN)

“I had to do a project with a male classmate and when I asked the teacher questions, the teacher always addressed my classmate, never me, he didn’t explain things to me.” (I14_Female OUT)

Some female participants experienced difficulties integrating with their classmates also:

“I have had experiences with male classmates of not speaking to me or to any of the other women in class until they realized that I had the best grades and then, suddenly I was a person with whom they wanted to talk a lot.” (I32_Female OUT)

“A friend of mine, XXX (female) was also very, very smart and she was very good at Engineering. So, our male classmates did not ignore her because she was intelligent and she helped a lot, but nevertheless, if they saw that you couldn’t contribute, they leave you aside.” (I20_Female OUT)

Even though female students who do not find any problems still feel outsiders in men’s networks. They do not feel they match the masculine interests, and struggle to get into a group. According to two respondents:

“Not only is it difficult to integrate into conversations outside class, but also in class, when groups are formed because of the things they have in common or simply because they are men … for a group project, one chooses friends (…) that closeness is difficult to have in the group or even the confidence to comment on things more calmly… for example, right now I have a project with four colleagues and several times they met to do part of the work among themselves. And they didn’t tell me anything … (…) Many times you don’t even feel like attending classes because you know what you’re going to find, the conversations they’re going to have, even small jokes….” (I07_Female IN)

“The way in which men relate to each other or the interests they may have outside of university are very different from what we (women) may have or the problems we may have. (…) For me it was not the same as being with my friends (women). Many times (when we met out of university) I went with a friend (female) because I did not feel comfortable.” (I06_Female IN)

And all of this affects the sense of belongingness. One participant shared the following statement referring to the competitiveness among men:

“I didn’t like that atmosphere of competition and comparison of grades.” (I20_Female OUT)

Others refer to the organizational system, as the university insists on the importance of changing your mind and thinking like an engineer, however some felt that they did not fit into that claim and that the system should change:

“I felt very alone, I felt that we had to know everything (…) I didn’t find any kind of support, no matter how much I asked for it.” (I21_MaleOUT)

“(…) I think that the system should change for those people who don’t fit in.” (I26_Female OUT)

In any case, both drop-out and non-drop out female students agree on the importance of having a good group of classmates at the university that supports you to continue your studies:

“At university I have a very good group of friends with whom I do my homework, study and attend classes. We help and support each other when we don’t understand something, an exam goes wrong or if we are missing some notes… It helps a lot to share worries, successes and failures. This makes studying and everyday life more enjoyable and easier.” (I05_Female IN)

“I met women and some guys with common interests and that helped me a lot. I think it is important to be comfortable at university. (…) in general, you must really want to persist and as much as you like a major, if you are alone, you feel alone, and it is very difficult for you to take it forward.” (I32_Female OUT)

#### Persistence

Analyzing the reasons that female and male students posit for dropping out, we found that there were important differences. Women tend to argue that they have faced mostly psychological pressure and emotional barriers.

“For me they were psychological barriers, because I left school with good grades and even though I was warned that Engineering was very hard, well I entered and basically except for the first exam, I failed everything. Even dedicating many hours and studying a lot, I felt that I was not able to go forward and that you need a huge capacity for sacrifice and being very smart to be able to graduate at the end.” (I17_Female OUT)

“You must expect the failure. This sounds very hard, but it’s even harder to get through your first exam having studied 8 hours a day and get a 2 or a 1. And sometimes we are not psychologically prepared for it, especially because in high school when we study, we pass and, in the university, it is not like that. What they do not tell you at the beginning of the major is that even having studied, you are going to fail.” (I16_Female OUT)

However, male students tend to cite their reasons for dropping out as not being sufficiently motivated, they never mentioned feeling they were not capable. In fact, men posit that they could cope with this high level of difficulty if they had made the effort. The problem for them was that the content was not what they have expected.

“The problem was the work that was going to be performed after graduation because Engineering is extremely difficult, but it’s nothing you can’t do, if you’re serious about it. But it’s just that so much work, so much effort to make a piece of metal.” (I23_Male OUT)

“I think it’s because of motivation, what I’m telling you is that it was difficult to find a way to start studying things that are not very attractive for most people. And realize that you don’t want to do that anymore. More than you can’t, it’s just that you don’t want to.” (I25_Male OUT)

#### Interventions or measures to pursue

Female interviewees who have persisted in Engineering shared different measures or recommendations that have helped them. Six of them refer to emotional support from family and friends:

“The support of your family, friends, people you trust, who encourage you to keep trying and not giving up, I think that in my case it has been the most important thing.” (I06_Female IN)

Seven students also mentioned the importance of having a support network within the university: friends and colleagues with whom you can share your problems, your failures and your successes. Students posit that this allows you to not feel alone and be constant and persistent, maintaining the pace of such a demanding major.

“At the university I have a very good group of friends with whom I do homework, study and go to class. We help and support each other when we don’t understand the subjects, an exam goes wrong or if we have missed some notes. It helps a lot to be able to share worries, successes and failures. This makes studying and everyday life more enjoyable and easier.” (I05_Female IN)

“The most important thing is to count on your classmates because we have done teamwork, asked questions and I think it is super effective because they are people who are available practically 24 hours a day, you can write them whenever you want and there is always a classmate who is super smart and will know how to solve any problem or you will be able to help others with something that they have not understood, that is, you will always find someone who will be able to help you.”(I27_Female IN)

Several personal qualities were also mentioned that can be helpful to succeed in Engineering. Among them, self-confidence was brought up by five students, while optimism and hope were other valuable qualities that were mentioned.

“It is very important, of course, not to lose hope, because this is a long-distance race and to hold on and finish it you need hope and to think that you can do it.” (I07_Female IN)

“I would tell them that they can do it and that they should not believe that the male student next to them in class is smarter or that he can do better than them.” (I09_Female IN)

### Quantitative results

In the interviews, we asked participants to quantify some of the nodes. Mann–Whitney U test was used to analyze differences in the mean values between male and female students who have dropped out (see Table 1) and between female students who persist and females who have dropped out (see Table 2).

**Table 1:** Mann–Whitney U Test on dropped out students (male vs. female).

	Gender		
	Male	Female	Mann–Whitney U Test
Goals	5.72	6.80	39.50
Curriculum perception	7.50	6.22	25.00
Self-efficacy perception	4.94	5.85	33.50
Sense of belongingness	2.44	4.22	29.50

**p < 0.01; *p < 0.05.

**Table 2:** Mann Whitney U Test on female students (non-drop out vs. drop out).

	Persistence in the major		
	Non-drop out	Drop out	Mann–Whitney U Test
Goals	4.63	6.80	37.00**
Curriculum perception	8.17	6.22	34.50**
Self-efficacy perception	3.90	5.85	47.00
Sense of belongingness	1.60	4.22	39.5*

**p < 0.01; *p < 0.05.

While comparing quantitative results from male and female students that have dropped out, no significant differences were found. Thus, even though it might seem that the main reasons for dropping out might have been similar, the underlying insights seem to have affected them differently. Whereas the comparison between female students who persist and females who have dropped out shows significant differences in goals, curriculum perception, and sense of belongingness.

Regarding goals, the results highlight that there are differences in the perception of gender role congruity perception between female students who drop out and those who do not. Female students withdrawing from Engineering find that there are greater differences between interests and aspirations (goals). Even having chosen this type of major at first, these women end up thinking that there are still different career paths for women and men. The question that arises is if they have always considered that there are careers more suitable for women or men or if the experience in a male-dominated major has led them to this perception.

Concerning curriculum perception, significant differences were found since non-drop-out female students seem to prefer practical and collaborative subjects. However, this can be explained by the fact that the interviewees state that this practical content is taught in the last years of the major. Therefore, students who have dropped out may not have had the opportunity to learn this practical content.

In the case of self-efficacy perception, no significant differences were found between self-efficacy perception for female students who persist and those who do not.

Lastly, in the case of sense of belongingness, marginal significance was found. Drop-out students found this climate much more difficult than their peers who have not dropped out. This is consistent with the qualitative findings as having a network of friends in class is mentioned as one of the key elements to persist.

## Conclusion and discussion

This research contributes to the literature (Wolffram et al., 2009; Sweeney, 2020; Mickelson et al., 2022) on amplifying Tinto’s persistence model from a gendered perspective. The findings of the present study make several important contributions to the existing literature on persistence in engineering majors, which can help future research and policies on this topic. Much of the previous research focused on reasons to persist without considering gender bias. Understanding how gender stereotypes and roles congruity affect female persistence can help to design better and more effective interventions.

Although Tinto’s model has received different critiques in the last years, with authors proposing its extension to include specific facets that might affect to concrete population (i.e., Botanga et al., 2021, with URM), our results support the theoretical model as a general framework to understand women drop out from Engineering when including the gender perspective. In this line, our results show that all the theoretical constructs proposed by the model (i.e., goals, curriculum perception, self-efficacy, sense of belongingness) work as (des)motivators in the expected way. Besides, although some results appear to be non-gendered (for instance, intrinsic motivators work better for both men and women to persist on the major), most of them are clearly gendered. In this line, most of the reasons of why women drop-out can be explained from the social role theory (Eagly, 1987a,b) as mainstreaming in each of the Tinto’s model constructs. Thus, no having contact with science or Engineer women that act as role models in early years affect female students both in establishing goals and in the sense of a low self-efficacy perception; the role-incongruity perception between being women (communal goals) and studying Engineering majors (agentic goals) affect not only to women themselves (increasing the impostor syndrome) but also to peer and teachers’ support, which increases the chilly environment and, in return, decrease their sense of belongingness. In this line, drop-out women still consider that there are still different career paths for women and men (different goals). Also, there are differences at the curricula, as female students who drop out do not find practice-oriented subjects or collaborative projects in the first course that could serve as mastery experiences, which leads to a decrease of their self-efficacy. Thus, in global, all this results in the fact that women tend to argue that they have faced mostly psychological pressure and emotional barriers, whereas men never mentioned it.

This research will allow to implement effective interventions to increase women’s persistence in engineering majors. The study advances our understanding of the barriers and obstacles that face female students in Engineering showing that motivation to persist is the result of multiple factors (i.e., goals, self-efficacy, curriculum, sense of belonging) that are affected by gender role perceptions. In the following sections, we will point out the most remarkable results and relate them with its practical implication in form of intervention proposals.

### Role congruency perception and role models

Our findings suggest that role congruency perception and lack of role models are more pronounced in female students who drop out. They also suggest that students know nearly nothing about the practical applications of Engineering when they enroll at university. Thus, it would be important to have interventions in early years, when gender stereotypes begin to affect expectations, interests and academic choices. Role models have been shown by extensive prior literature to be critical in motivating students to follow a path and achieve goals (Collins, 1996; Lockwood and Kunda, 1997). However, there is no unanimous agreement on how these models should be. Certainly, they must be seen as competent and successful by female students (Marx et al., 2013). Likewise, it is essential that they can feel identified, so they must belong to the same gender and ethnicity (Lockwood, 2006). Regarding lack of knowledge about the content of Engineering and its practical application, we recommend holding sessions in high schools for both female and male students (Falco and Summers, 2019). These sessions could be held by current Engineering female students, because it will be easier for high schoolers to identify with them (Mussweiler, 2003). Relating to the content, we propose that these role models focus on how these professions can contribute to society, showing innovations congruent with communal goals led by female engineers (Boucher et al., 2017). It is important to highlight that these professions solve real problems, can help others and improve our lives (González-Pérez et al., 2020). We recommend holding these interventions for both female and male students as it is also important for male students to dispel gender stereotypes. Nevertheless, these sessions could also be held by men not conforming to agentic masculine stereotypes, which could help women and girls to see them as allies (Cheryan et al., 2011). Furthermore, university summer sessions for high schoolers could be a game-changing early experience to boost interest in Engineering (Kitchen et al., 2018). To reduce these gender stereotypes, it could be interesting to have informative sessions with high school teachers and families to broaden their minds, as parents and teachers become principal role models, advisors and supporters for females’ academic choices (Gunderson et al., 2012). Furthermore, we suggest inviting career advisors at high school level, as they could unbiasedly help students to find what they are good at and what career paths are more suitable for them regarding their interests and strengths (Falco, 2017).

### Self-efficacy perception

A second conclusion of our research is that female students, regardless of whether they have dropped out or not, tend to have lower self-efficacy perceptions than males, which can lead them to drop out. Female students often self-impose higher standards of excellence, making them believe that they are not capable of persisting. In a context where women are a minority and, as noted, face multiple barriers, extensive previous research has identified self-efficacy as a key predictor of women’s success in engineering (Blaisdell, 2000; Marra et al., 2005). To promote self-efficacy perception, it is worthwhile focusing on the self-efficacy sources proposed by Bandura (1997). So, following Betz (2004), we propose intervention programs that would include: (1) activities in which they can recognize that they are/were successful (mastery experiences), (2) interaction with female role models (both professionals and recent students) through mentorship programs (social modelling), (3) professors focusing on students’ success and capability to perform difficult tasks (verbal persuasion), and (4) promoting positive emotions through both interventions to foster a growth mindset highlighting that intelligence and ability are not fixed traits and positive psychological programs (emotional and physiological states) to positively influence their criteria to judge their capability and vulnerability. These interventions can help to demonstrate that hard work and effort can help female students to overcome challenges, and that they are able to do it.

### Sense of belongingness

Sense of belongingness becomes a key predictive factor in terms of persistence (Walton et al., 2015). According to our results, both drop-out and non-drop out female students agree on the importance of having a group of classmates at university and a family that support you to continue your studies. Literature has identified that the sense of belonging is related to the skills to make an effort in the face of difficulties (Vaz et al., 2015). These skills become even more relevant in a context such as that of engineering students who need to overcome important barriers and, on many occasions, feel alone and without support (Strayhorn, 2018). On the one side, having a strong supportive network of peers motivates female students to not drop out (Limbert, 1995). Therefore, we propose mentorship programs within the university where freshman engineering students are mixed with sophomore, junior or senior engineering students (Packard, 1999) with the aim of retaining female students through a nurturing mentoring program, designed to build a network with other female Engineering students with whom they can easily identify (Stout et al., 2011). Thus, these programs will enhance personal support through contacts with peer female role models, will build confidence and self-efficacy as mentees will see that their mentors have been able to succeed in Engineering and provides valuable emotional support. This mentorship program can also involve collaboration between university students and networks of engineers, in order to help seniors in their immersion in the professional workplace. Another intervention could be peer-led team (Horwitz et al., 2009) learning to provide female students with an efficient and supportive study group, where through workshops, a coached student who has previously been successful in the course facilitates learning. It could also be interesting to promote interventions with male and female students to highlight the importance that diversity has for innovation, promoting mix-gendered groups. Finally, student-run clubs and initiatives can also enhance a sense of belonging (Sahin, 2013).

On the other side, as it is crucial for a sense of belongingness to feel encouraged and motivated by the faculty, the Gender Compliance Committee or Diversity and Inclusion Dean should have more significance. Communications, performance and language to detect gender stereotypes need to be carefully reviewed to mitigate gender stereotypes (Cheryan et al., 2011). Following Blickenstaff (2005), course materials and assignments should also be reviewed to add female scientists and their achievements to shift perceptions about who belongs, while promoting diversity-related activities. Furthermore, training or workshops with the faculty to foster some self-reflection, review performance, identify gender bias and implement solutions could be another interesting intervention.

### Curriculum perception

Another finding of our research are the masculine biases in the curriculum that sometimes prevent female students from persisting. Its content is adapted to the interests and perspectives of both the teacher and the dominant social group in the class, or both (Beder, 1989; Lewis, 1995). This leads to a new difficulty for engineering students since, since most of the faculty and engineering students are men, they may feel uncomfortable or excluded in class. In short, the content of the curriculum becomes a new barrier that can lead students to drop out, switch or not succeed in their majors. Therefore, we propose active learning and project-based instruction using collaboration techniques from the first years (Zastavker et al., 2006; Dominguez et al., 2019), to enhance a sense of community rather than a competitive environment. Incorporating service-learning projects to promote the idea that Engineering helps to improve society and allowing more choice in terms of subjects could boost interest and motivation. These new subjects could be more focused on social purposes, environmental impact or sustainability ethics that connect better with communal goals (Diekman et al., 2015).

Apart from the curriculum itself, our research found masculine biases in the grading systems. We suggest increasing collaboration rather than competitiveness. Instead of multiple-choice tests or exams where only the final answer matters, we propose replacing them with open-ended evaluations. A constructive response system that allows students to show their competence through writing has been proved to be more suitable for female students (Weaver and Raptis, 2001).

## Conclusion

Finally, as a general intervention for fostering persistence, we suggest highlighting the importance of building soft skills, as mentioned before in the case of self-efficacy. Self-efficacy, hope, resilience and optimism (positive psychological capital; Luthans et al., 2007) are qualities that can help female students to overcome the obstacles and barriers they will find in their academic and professional careers. On the other hand, having counselling and psychological services could be a useful tool to reduce stress, anxiety and depression among female students.

In conclusion, based on the findings of this research, including a gendered perspective in Engineering fields provide a promising route to retaining female students. With our empirical results, we have been able to validate our proposed theoretical framework and build upon each of the parts of Tinto’s well-known validated theoretical model of persistence, incorporating this gender perspective. Women who drop out of Engineering highlight in the interviews that goals incongruity leads them to low levels of motivation, affecting persistence. The results show that this lack of congruity influences mainly belongingness and self-efficacy perception. There are several practices that institutions should revisit and rethink to provide the necessary support to Engineering female students who are struggling. According to the gender differences outlined in this research, we cannot understand women’s persistence in engineering without a gender perspective. Women enter male-dominated majors where they do not feel as if they belong; for instance, they stated that they are more comfortable responding to praise than to challenge. We have found that including these communal goals for real could improve retention in these majors, as they could be seen as congruent with their priorities. Findings from the present study allow policymakers and organizations to implement interventions which encourage female student persistence in male-dominated fields. Providing women with a strong support system can help them to prevail over barriers which they may face during their Engineering education. All these measures should be accompanied by a learning and social environment that promotes the reduction of gender stereotypes (Solbes-Canales et al., 2020), so the next generation of potential female engineers believe that they will be successful.

### Limitations and directions for future research

These results are based on a limited sample of female engineers who have dropped out. A larger sample would be desirable, especially to strength the quantitative analysis with more robust methodologies. It would also be interesting to delve into whether the reasons are common to all engineering disciplines in general or are limited to some specific ones.

The findings from the present study suggest other promising directions for future research, for example to carry out a longitudinal study. Understanding what kinds of barriers students face at different points in their careers can provide a more comprehensive view and help design effective measures.

## Data availability statement

The raw data supporting the conclusions of this article will be made available by the authors, without undue reservation.

## Author contributions

SG-P, MM, VP, and EC-G designed, performed, and analyzed the research, searched the literature, and wrote the manuscript. All authors contributed to the article and approved the submitted version.

## Funding

This research has received grant PID2020-114183RB-I00, funded by MCIN/AEI/10.13039/501100011033. This research has received financial support from: Cátedra Universidad CEU San Pablo and Mutua Madrileña, Spain (060516-USPMM-03/18) and from Karmo Spirit S.L (Ref.: 001). The funders were not involved in the study design, collection, analysis, interpretation of data, the writing of this article or the decision to submit it for publication.

## Conflict of interest

The authors declare that the research was conducted in the absence of any commercial or financial relationships that could be construed as a potential conflict of interest.

## Publisher’s note

All claims expressed in this article are solely those of the authors and do not necessarily represent those of their affiliated organizations, or those of the publisher, the editors and the reviewers. Any product that may be evaluated in this article, or claim that may be made by its manufacturer, is not guaranteed or endorsed by the publisher.

## Acknowledgments

We acknowledge the generosity of Real Academia de Ingenieria and ASTI Talent and Technology Foundation without which the present study could not have been completed. We are also grateful to Professor Ruth Mateos de Cabo for their valuable comments and suggestions.

## Supplementary material

The supplementary material for this article can be found online at: https://www.frontiersin.org/articles/10.3389/fpsyg.2022.918439/full#supplementary-material
