# Supplementary material for: I am done with this! Women dropping out of engineering majors
Source: Front Psychol. 2022 Aug 12;13:918439. doi: 10.3389/fpsyg.2022.918439 (PMC9415617; doi:10.3389/fpsyg.2022.918439)
Supplement: Supplementary file 1 [file Table_1.docx]

Table 1. Participants’ characteristics

|  | YEAR OF BIRTH | CURRENT MAJOR | CURRENT UNIVERSITY | PREVIOUS DEGREE | PREVIOUS UNIVERSITY |
| --- | --- | --- | --- | --- | --- |
| I01_FEMALE_In | 1997 | Physical Engineering | Public | --- | --- |
| I02_FEMALE_ In | 1999 | Industrial and Automatic Electronic Engineering | Public | Industrial Technologies Engineering | Public |
| I03_FEMALE_ In | 2001 | Food Engineering | Public | --- | --- |
| I04_FEMALE_ In | 2001 | Food Engineering | Public | --- | --- |
| I05_FEMALE_ In | 2000 | Industrial Technologies Engineering | Public | --- | --- |
| I06_FEMALE_ In | 1999 | Informatics Engineering + Business Administration | Private | --- | --- |
| I07_FEMALE_ In | 2000 | Informatics Engineering + Business Administration | Private | --- | --- |
| I08_FEMALE_ In | 1999 | Industrial Technologies Engineering | Private | --- | --- |
| I09_FEMALE_ In | 1997 | Industrial Technologies Engineering | Public | --- | --- |
| I10_FEMALE_ In | 1998 | Industrial Technologies Engineering | Private | --- | --- |
| I11_MALE_Out | 1999 | Business Administration + Economy | Private | Mechanical Engineering | Private |
| I12_MALE_Out | 1997 | Economy | Private | Informatics Engineering | Public |
| I13_MALE_Out | 1997 | Business Intelligence | Private | Civil Engineering/ Informatics Engineering | Public/ Private |
| I14_FEMALE_Out | 1999 | Human Nutrition and Dietetics | Private | Telecommunication Engineer | Public |
| I15_MALE_Out | 1999 | Business Administration | Private | Industrial Technologies Engineering | Private |
| I16_FEMALE_Out | 2003 | Business Administration | Private | Telecommunication Engineer | Private |
| I17_FEMALE_Out | 1997 | Economy | Private | Industrial Technologies Engineering | Private |
| I18_FEMALE_Out | 2001 | International Business Administration | Private | Informatics Engineering+ Business Administration | Private |
| I19_FEMALE_In | 2000 | Informatics Engineering | Private | --- | --- |
| I20_FEMALE_Out | 1998 | Business Administration (bilingual) | Private | Industrial Technologies Engineering | Private |
| I21_MALE_Out | 1995 | Architecture | Public | Aerospace Engineering | Public |
| I22_MALE_Out | 1998 | Business Intelligence | Private | Telecommunication Engineer | Public |
| I23_MALE_Out | 1994 | Architecture | Public | Aerospace Engineering | Public |
| I24_FEMALE_Out | 1999 | Psychology | Public | Chemical Engineering | Public |
| I25_MALE_Out | 2001 | Business Administration | Private | Telecommunication Engineer | Private |
| I26_FEMALE_Out | 2002 | Business Intelligence | Private | Telecommunication Engineer | Private |
| I27_FEMALE_In | 2000 | Industrial Technologies Engineering | Private | --- | --- |
| I28_FEMALE_Out | 1997 | Business Administration (English) | Private | Industrial Technologies Engineering | Public |
| I29_FEMALE_Out | 1996 | 3D Animation | Private | Audio-visual Systems Engineering | Public |
| I30_FEMALE_In | 2003 | Forest Engineering | Public | --- | --- |
| I31_FEMALE_In | 2000 | Industrial Organization Engineering | Public | --- | --- |
| I32_FEMALE_Out | 1993 | Communication Electronics Engineering | Public | Physical Engineering |  |
| I33_FEMALE_In | 2003 | Forest Engineering | Public | --- | --- |
| I34_MALE_Out | 1997 | Economy + International Relationships | Private | Telecommunication Engineering | Public |
