# Supplementary material for: I am done with this! Women dropping out of engineering majors
Source: Front Psychol. 2022 Aug 12;13:918439. doi: 10.3389/fpsyg.2022.918439 (PMC9415617; doi:10.3389/fpsyg.2022.918439)
Supplement: Supplementary file 2 [file Table_2.docx]

Table 2. Definitions of nodes

|  | **DEFINITIONS OF NODES** |
| --- | --- |
| **Gender Congruity** | Ingrained stereotypes in society negatively affect the choice of a STEM major (Eagly, 1987a). |
| **Goals** | **Attitude and early experiences**  Girls enjoy their science experiences in school but have strong negative feelings about dissection and cannot imagine themselves as scientists. The girls also notice the bias in textbooks and television where very few scientists are depicted as female (Baker and Leary, 1995). |
|  | **Role models**  A low proportion of women in a discipline probably sends a message to girls that the discipline is unattractive to women, and they should avoid it too (Blickenstaff, 2005).  Family role models have also been an important source of self-efficacy for women in STEM. The proximal presence of an engineer can increase one’s perceived likelihood of success and satisfaction in the field (Koul et al., 2011; Zeldin and Pajares, 2000). |
|  | **SES (Socio Economic Status)**  Highly educated parents and high socioeconomic status have significant influence on women’s STEM course choices. It is possible that highly educated parents have less conventional beliefs about appropriate career choices for females and are consequently more willing to encourage their daughters in non-traditional pursuits (Ware et al., 1985). |
| **Self-efficacy perception** | Self-efficacy is concerned with judgements about how well one can organize and execute courses of action required to deal with prospective situations containing many ambiguous, unpredictable and often stressful elements (Bandura, 1977, 1997).  Women’s lower self-efficacy perception in the STEM field is related to the fact that women generally judge themselves as being less suitable or unable to obtain good marks in some subjects. |
| **Sense of Belongingness** | The feeling of belongingness is often constrained by subtle cues in achievement environments that signal who naturally belongs there and is most likely to succeed and who else is a dubious fit (Dasgupta, 2011).  **Chilly climate with classmates**  A chilly climate with classmates acts as a barrier blocking the route to their degrees, including feelings of isolation and intimidation, sexual harassment as well as a loss in self-confidence as they progress through their major program (Blickenstaff, 2005).  **Chilly climate with teachers**  Chilly climate with teachers includes student-teacher interactions in which teachers treat girls in a derogatory or discriminatory way (Blickenstaff, 2005; Kelly, 2016). |
| **Curriculum perception** | Harsh, competitive grading systems, densely packed curricula, and a lack of teaching for conceptual understanding (Zohar and Sela, 2003; Seymour, 1995) negatively affects women undergraduates in STEM. Whereas hands-on tasks, employing active learning techniques, communal, collaborative learning environments and teaching an understanding of the social relevance of subjects in their everyday worlds has a positive impact on self-efficacy (Jansen et al., 2015). |
| **Persistence** | This node includes all arguments provided by the participants to drop out. |

**References:**

Baker, D., and Leary, R. (1995). Letting girls speak out about science. *J. Res. Sci. Teach.* 32, 3–27. doi: 10.1002/tea.3660320104

Bandura, A. (1977). Self-efficacy: toward a unifying theory of behavioral change. *Psychological review*, *84*(2), 191.

Bandura, A. (1997). *Self-Efficacy: The Exercise of Control*. New York: W H Freeman & Co.

Blickenstaff, J. C. (2005). Women and science careers: leaky pipeline or gender filter? *Gend. Educ.* 17, 369–386. doi: 10.1080/09540250500145072

Dasgupta, N. (2011). Ingroup experts and peers as social vaccines who inoculate the self-concept: the stereotype inoculation model. *Psychol. Inq.* 22, 231–246. doi: 10.1080/1047840X.2011.607313

Eagly, A. H. (1987a). *Sex Differences in Social Behavior: A Social-Role Interpretation*. New York: Lawrence Erlbaum Associates, Inc.

Jansen, M., Scherer, R., and Schroeders, U. (2015). Students’ self-concept and self-efficacy in the sciences: differential relations to antecedents and educational outcomes. Contemp. Educ. Psychol. 41, 13–24. doi: 10.1016/j.cedpsych.2014.11.002

Kelly, A. M. (2016). Social cognitive perspective of gender disparities in undergraduate physics. *Phys. Rev. Phys. Educ. Res.* 12:020116. doi: 10.1103/PhysRevPhysEducRes.12.020116

Koul, R., Lerdpornkulrat, T., and Chantara, S. (2011). Relationship between career aspirations and measures of motivation toward biology and physics, and the influence of gender. *J. Sci. Educ. Technol.* 20, 761–770. doi: 10.1007/s10956-010-9269-9

Seymour, E. (1995). Guest comment: why undergraduates leave the sciences. *Am. J. Phys.* 63, 199–202. doi: 10.1119/1.17954

Ware, N. C., Steckler, N. A., and Leserman, J. (1985). Undergraduate women: who chooses a science major? *J. High. Educ.* 56, 73–84. doi: 10.1080/00221546.1985.11778705

Zeldin, A. L., and Pajares, F. (2000). Against the odds: self-efficacy beliefs of women in mathematical, scientific, and technological careers. *Am. Educ. Res. J.* 37, 215–246. doi: 10.3102/00028312037001215

Zohar, A., and Sela, D. (2003). Her physics, his physics: gender issues in Israeli advanced placement physics classes. *Int. J. Sci. Educ.* 25, 245–268. doi: 10.1080/09500690210126766
