# Supplementary figures and images for: I am done with this! Women dropping out of engineering majors
Source: Front Psychol. 2022 Aug 12;13:918439. doi: 10.3389/fpsyg.2022.918439 (PMC9415617; doi:10.3389/fpsyg.2022.918439)

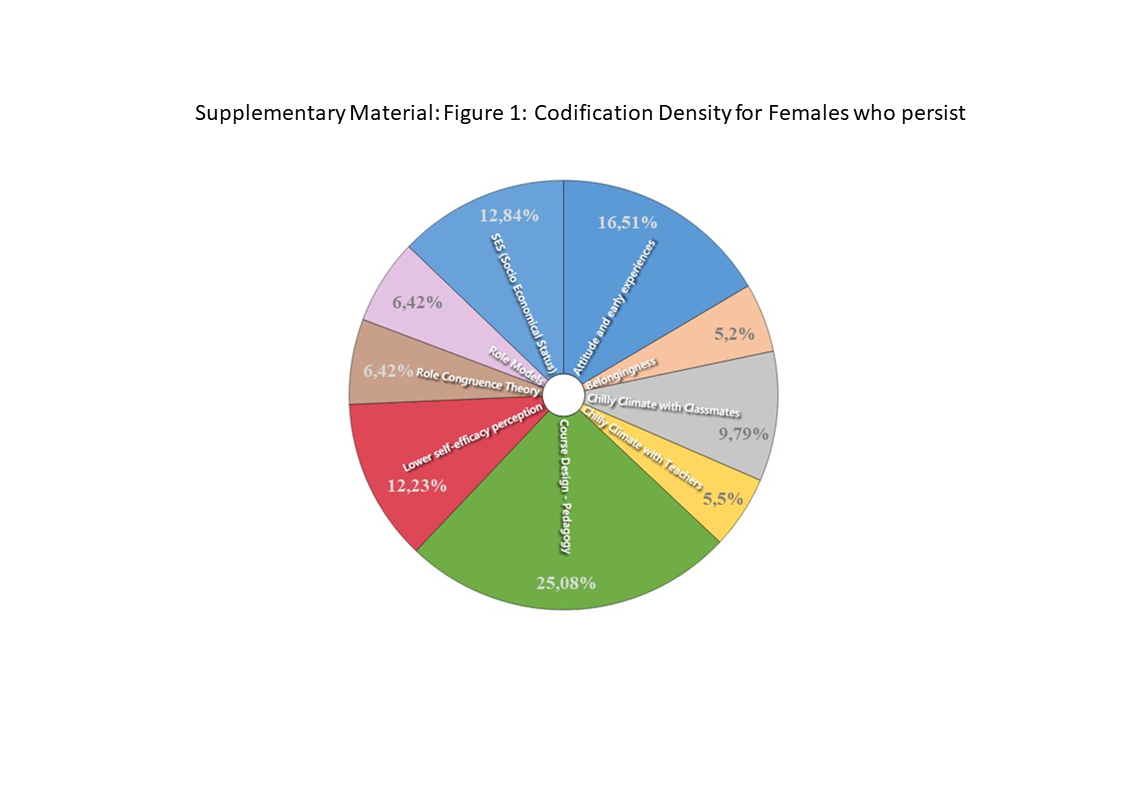

Supplement: Supplementary file 3 [file Image_1.PNG]

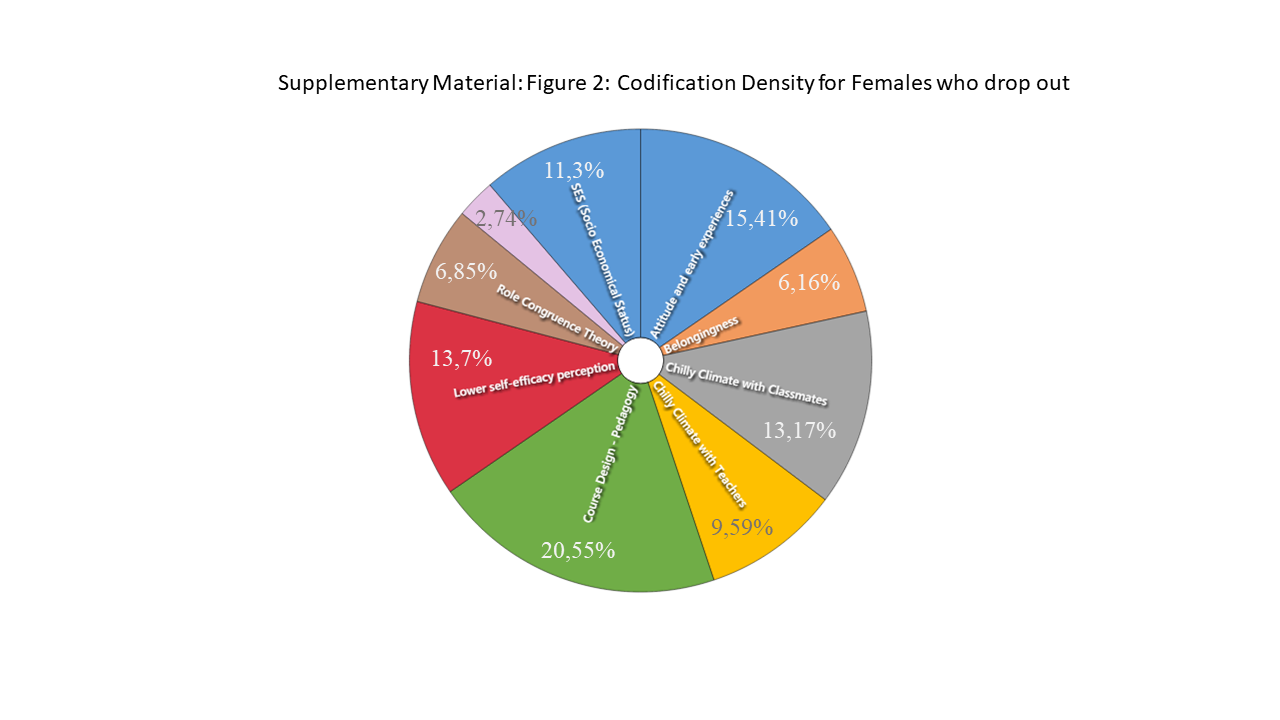

Supplement: Supplementary file 4 [file Image_2.PNG]

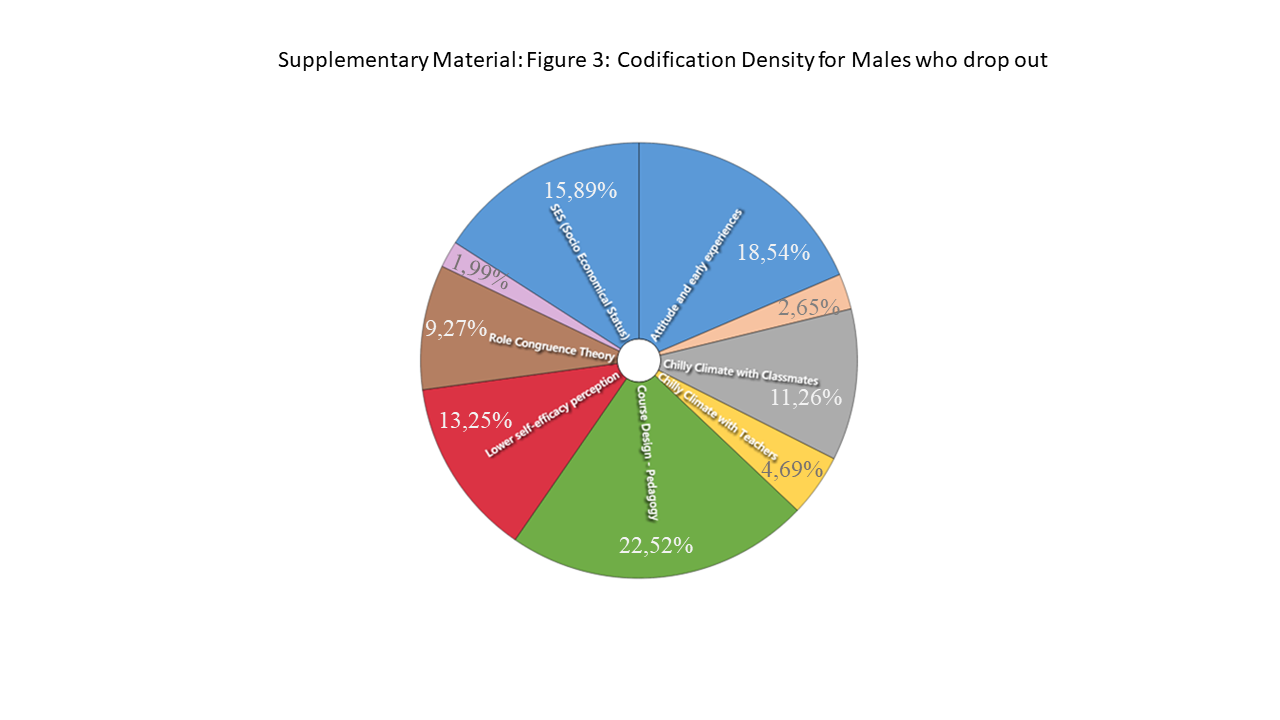

Supplement: Supplementary file 5 [file Image_3.PNG]
